# Modified lateral approach combined with medial percutaneous approach versus triceps tongue-shaped flap approach and bilateral triceps brachii approach for pin fixation in treatment of irreducible displaced pediatric supracondylar humeral fractures

**DOI:** 10.1097/MD.0000000000035158

**Published:** 2023-09-08

**Authors:** Hu Da, Liang Zhou, Qiao-Yun Xi, Chang-Ming Xu

**Affiliations:** a Department of Orthopaedics, Lianshui County People’s Hospital, Lianshui, China.

**Keywords:** bilateral triceps approach, children, modified lateral approach combined with medial percutaneous approach, pin fixation, supracondylar humeral fracture, triceps tongue-shaped flap approach

## Abstract

To evaluate the clinical outcomes of the modified lateral approach combined with the medial percutaneous approach (MLACMPA) versus the triceps tongue-shaped flap approach (TTSFA) and the bilateral triceps brachii approach (BTBA) in the treatment of irreducible displaced supracondylar humeral fractures (SHFs) in children. Between March 2000 and July 2022, a total of 135 children who underwent open reduction and Kirschner wire cross internal fixation for irreducible displaced SHFs caused by trauma were retrospectively analyzed. According to the surgical approach, the patients were assigned to the TTSFA group (n = 36), the BTBA group (n = 40) and the MLACMPA group (n = 59). The duration of surgery, intraoperative blood loss, incision length, and elbow range of motion were compared. The 3 groups were similar in terms of mean age, sex distribution, and time from injury to operation. The duration of surgery, intraoperative blood loss, incision length and postoperative elbow range of motion in the MLACMPA group were significantly superior to those in the TTSFA group and BTBA group (*P *< .05). Compared the use of the TTSFA or the BTBA, using the MLACMPA for pin fixation in the treatment of irreducible displaced pediatric SHFs could significantly shorten the duration of surgery, reduce the operation trauma, facilitate earlier functional exercise of joints after operation and yield better elbow function.

## 1. Introduction

Supracondylar fractures of the humerus are common fractures in children.^[1,2]^ In most cases, good results can be obtained by closed reduction.^[3]^ However, closed reduction attempts do not yield satisfactory alignment in some supracondylar fractures of the humerus that constitute a small proportion of fractures classified as Gartland III. Patients with these fractures have to undergo surgical treatment.^[1,4]^ The rate of open reduction in our study period was approximately 4%. The most classical surgical approach is the triceps tongue-shaped flap approach (TTSFA).^[5]^ A longitudinal incision is made in the posterior median surface of the elbow, followed by a wide separation between the deep and superficial fascia. In particular, the ulnar nerve needs to be isolated for protection. When the triceps tendon is cut in the lingual (inverted V) shape and turned downward, the distal humerus will be exposed. With this surgical approach, the fracture site can be well exposed.^[6]^ However, the integrity of the triceps brachii is destroyed, and early functional training for the elbow joint is difficult.^[7]^ In addition, scar adhesion of the soft tissue around the joint, muscle fibrosis, and joint capsule as well as surrounding ligament contraction and myositis ossification occurred and eventually led to joint stiffness.^[1,8,9]^

To protect the triceps tendon, some authors have improved the surgical approach with the bilateral triceps brachii approach (BTBA).^[10]^ The posterior median incision of the elbow is used, and then a wide separation between the deep fascia and superficial fascia is made. After the ulnar nerve is exposed and protected, the muscular space at the front edge of the medial and lateral triceps muscles is separated, and the fracture site can be reached. The BTBA has only 1 incision at the back of the elbow joint, and the integrity of the brachial triceps tendon is protected. The extension and flexion function of the elbow joint can be exercised early.^[11]^ However, in this surgical approach, the range of separation between the superficial fascia and the deep fascia at the back of the elbow joint is substantially larger than that of TTSFA, and there is still considerable trauma. In addition, the exposure range of fracture site is less, which increases the difficulty of reduction. It was found in the clinic that some children still experienced complications such as scar adhesion of periarticular soft tissue and stiffness of the elbow joint after operation.^[12]^

To minimize soft tissue damage to the elbow joint and obtain good elbow function, some authors have further improved the surgical approach. A modified lateral approach combined with medial percutaneous approach (MLACMPA) was used to treat irreducible displaced pediatric supracondylar humeral fractures (SHFs).^[13]^ The purpose of this study is to clarify the efficacy of the MLACMPA by comparing it with the TTSFA and the BTBA in terms of the duration of surgery, the amount of intraoperative bleeding, the length of surgical incision, and the postoperative elbow range of motion.

## 2. Methods

### 2.1. Research object

Data from a total of 135 children who underwent open reduction and Kirschner wire cross internal fixation for irreducible displaced SHFs caused by trauma were retrospectively analyzed. Between March 2000 and June 2006, 36 children underwent surgery with TTSFA. Between July 2006 and August 2014, 40 children underwent surgery with the BTBA. Between September 2014 and July 2022, 59 children underwent surgery with the MLACMPA. All methods were carried out in accordance with the relevant guidelines and regulations. The authors had access to information that could identify individual participants during data collection. All patient details have been deidentified. This study was approved by the Ethics Committee of the Lianshui County People Hospital, and written informed consent was obtained from all guardians of the children. The reporting of this study conforms to the CONSORT guidelines.^[14,15]^ Fellow researchers may reproduce our methodology from the description given in this methods section.

### 2.2. Inclusion and exclusion criteria for the research

The inclusion criteria were as follows: all cases were Gartland type III fractures, in whom adequate closed reduction could not be achieved; The age range was 4 to 9 years; and surgical treatment was performed within 7 days after injury. The exclusion criteria were as follows: neurovascular compromise; comminuted intercondylar fracture of the humerus; The patients did not perform adequate elbow joint exercise after surgery; and patients lacked outpatient follow-up after discharge, or the follow-up time was <6 months.

### 2.3. Experimental grouping

According to the surgical approach, the patients were assigned to the TTSFA group (n = 36), BTBA group (n = 40) and MLACMPA group (n = 59), rather than procedures based on the preference of the surgeon.

### 2.4. Surgical technique

TTSFA and BTBA have been introduced in the introduction section. The following describes the MLACMPA in detail. Under general anesthesia, the patients were placed in a supine position with their elbows close to the edge of the operating table. Two surgeons were involved in each operation. A modified lateral elbow joint incision was chosen in the MLACMPA. An incision approximately 4 to 6.5 cm long is started from the intersection point of the transverse lines on the cubital fossa and the lateral side of the upper arm, extending backward and downward in an arc and ending at the lateral edge of the olecranon of the ulna. After the deep fascia is cut, the proximal end of the incision is cut along the space between the triceps brachii and the brachioradialis muscle to the periosteum of the humerus, which can expose the bone crest on the external side of the fracture end. At the far end of the incision, a small portion of the insertion point of the triceps brachii aponeurosis is cut off on the posterior joint capsule of the elbow, and then the triceps brachii is pulled back and down. In this way, the posterior side of the fracture can be fully exposed, including the lateral column, the olecranon fossa and the medial column. When the brachioradialis muscle is pulled forward, the front side of the lateral humerus can be exposed. Finally, the bone crest at the lateral epicondyle of the humerus, the anterior lateral surface of the distal humerus, the olecranon fossa, the lateral column, and a part the medial column are exposed, and only the medial surface of the fracture end are not exposed. However, this is enough for reduction of the fracture. Under direct vision, surgeons can perform reduction and Kirschner wire internal fixation. Under C-arm guidance, when it is confirmed that the fracture has been well reduced, percutaneous internal fixation of the medial epicondyle of the humerus with a Kirschner wire can be performed. The medial epicondyle of the humerus and the ulnar nerve groove should be carefully touched and distinguished, the percutaneous Kirschner wire is inserted into the most prominent point on the medial side of the elbow joint, and drilling from the anteromedial to the posterolateral is performed. It is particularly important to clearly identify the ulnar nerve groove to ensure that the Kirschner wire will not be placed in the ulnar nerve groove and cause damage to the ulnar nerve. Then, the Kirschner wire is bent at 90° to prevent migration.^[11]^ Finally, the elbow is fixed externally in the functional position with plaster. Immediately postoperatively, neurological assessment of the ulnar nerve is performed. The above elbow plaster is removed after 2 weeks, allowing early progressive active and active-assisted elbow range of motion (ROM) with wires in place. Once healing is radiologically detected, usually between 4 and 6 weeks, the Kirschner wires are removed. Follow-up visits are generally scheduled for 1 week, 1 month, 2 months, 4 months and 6 months following discharge.

### 2.5. Elbow range of motion

After we removed the plaster, the patients performed elbow extension and flexion exercises under the guidance of the rehabilitation physician.^[16–18]^ At 2, 4, and 6 months postoperatively, the ROMs of both elbows were assessed by a goniometer.

### 2.6. Other data

Data regarding the duration of surgery, intraoperative blood loss and incision length were obtained precisely and recorded in detail. All the above results were analyzed by the same surgeon, and this individual was completely unaware of the grouping and treatment methods.

### 2.7. Statistical analysis

SPSS statistics 24.0 (IBM, Armonk, NY) was used for statistical analysis. All data are expressed as the mean ± standard deviation. Data from all groups were analyzed statistically. The chi-square test was used to compare categorical variables between the groups, while an independent *t* test was used to compare numerical variables between the groups. Before an independent *t* test was applied, the data were checked and confirmed to be approximately normally distributed. *P* values of < .05 (*P* < .05) were considered statistically significant.

## 3. Results

In the TTSFA group, 27 patients were male, and 9 patients were female, with a mean age of 6.71 ± 1.16 years (range, 4.22–8.91 years). In the BTBA group, 29 patients were male, and 11 patients were female, with a mean age of 6.08 ± 1.00 years (range, 4.12–8.57 years). In the MLACMPA group, 39 patients were male, and 20 patients were female, with a mean age of 6.33 ± 0.98 years (range, 4.03–8.98 years). In the TTSFA group, 10 patients had fractures on the left side and 26 on the right. In the BTBA group, 12 patients had fractures on the left side and 28 on the right. In the MLACMPA group, 17 patients had fractures on the left side and 42 on the right. The mean time from injury to the definitive surgical procedure was 2.01 ± 1.05 days in the TTSFA group (range, 0.82–5.87 days), 2.09 ± 0.99 hours in the BTBA group (range, 1.11–6.18 days), and 2.23 ± 1.02 days in the MLACMPA group (range, 1.21–6.51 days). There were no significant differences in sex, age or time from injury to operation between the MLACMPA group and the TTSFA and BTBA groups (*P* > .05), indicating comparability. Demographic and clinical characteristics of the patients are shown in Table 1. No patient in either group experienced vascular or nerve injuries, a major loss of reduction, fracture nonunion or pin-tract infection.

**Table 1:** Demographic and clinical characteristics of patients.

	TTSFA group (n = 36)	BTBA group (n = 40)	MLACMPA group (n = 59)	*P*	
				TTSFA group/MLACMPA group	BTBA group/MLACMPA group
Age (yr)					
Range (min–max)	4.22–8.91	4.12–8.57	4.03–8.98	.09	.21
Mean ± SD	6.71 ± 1.16	6.08 ± 1.00	6.33 ± 0.98		
Sex					
Male	27	29	39	.87	.79
Female	9	11	20		
Side					
Right	10	12	12	.55	.42
Left	26	28	47		
Injury/surgery interval (d)					
Range (min–max)	0.82–5.87	1.11–6.18	1.21–6.51	.31	.48
Mean ± SD	2.01 ± 1.05	2.09 ± 0.99	2.23 ± 1.02		

BTBA = bilateral triceps brachii approach, MLACMPA = modified lateral approach combined with medial percutaneous approach, TTSFA = triceps tongue-shaped flap approach.

Data regarding the duration of surgery, intraoperative blood loss and incision length in all groups are detailed in Table 2. All results of the MLACMPA group were significantly superior to those of the TTSFA group and the BTBA group (*P *< .05).

**Table 2:** Intra-operative data.

	TTSFA group (n = 36)	BTBA group (n = 40)	MLACMPA group (n = 59)	*P*	
				TTSFA group/ MLACMPA group	BTBA group/MLACMPA group
Duration of surgery (min)					
Range (min–max)	38–111	35–81	21–71	<.01	<.01
Mean ± SD	58 ± 18	48 ± 12	29 ± 12		
Intra-operative blood loss (mL)					
Range (min–max)	50–205	40–155	20–100	<.01	<.01
Mean ± SD	98 ± 33	76 ± 25	51 ± 19		
Incision length (cm)					
Range (min–max)	6.9–10.1	7.6–11.5	4.2–6.4	<.01	<.01
Mean ± SD	8.4 ± 0.9	9.3 ± 1.1	5.0 ± 0.6		

BTBA = bilateral triceps brachii approach, MLACMPA = modified lateral approach combined with medial percutaneous approach, TTSFA = triceps tongue-shaped flap approach.

The elbow ROMs at months 2, 4, and 6 post-operation are presented in Table 3. The results showed that the elbow ROM in the MLACMPA group was significantly greater than that in the TTSFA and BTBA groups at all postoperative time points (*P *< .05).

**Table 3:** Elbow ROM (º).

	TTSFA group (n = 36)	BTBA group (n = 40)	MLACMPA group (n = 59)	*P*	
				TTSFA group/ MLACMPA group	BTBA group/MLACMPA group
Elbow ROM at mo 2					
Range (min–max)	45–110	60–120	65–130	.01	.02
Mean ± SD	80 ± 20	82 ± 16	90 ± 17		
Elbow ROM at mo 4					
Range (min–max)	75–125	80–130	90–140	<.01	.03
Mean ± SD	88 ± 13	96 ± 12	105 ± 14		
Elbow ROM at mo 6					
Range (min–max)	80–140	100–140	120–140	<.01	<.01
Mean ± SD	113 ± 16	123 ± 12	134 ± 7		

BTBA = bilateral triceps brachii approach, MLACMPA = modified lateral approach combined with medial percutaneous approach, ROM = range of motion, TTSFA = triceps tongue-shaped flap approach.

## 4. Discussion

The degree of soft tissue injury in surgery is one of the important factors that determine the recovery of elbow function.^[19,20]^ For the elbow, the biggest benefit of reduced trauma in surgery is that the degree of soft tissue adhesion and contracture after surgery is less, and good extension and flexion function can be obtained.^[21]^

For pediatric SHFs, the classical surgical approach is the TTSFA.^[5]^ It has the advantages that the fracture can be fully exposed, which is convenient for the reduction and internal fixation of the fracture. The disadvantage is that the trauma of the operation is large, the continuity of the triceps brachii is destroyed, and the amount of intraoperative bleeding and the operation time is increased. After the operation, the elbow cannot be exercised at an early stage, the joint capsule is prone to contracture, and the scar is often adhered, resulting in difficulties regarding extension and flexion of the elbow.^[22]^

The BTBA is improved on the basis of the TTSFA.^[10]^ With the BTBA, the integrity of the triceps brachii can be protected, and elbow extension and flexion can be performed early after the operation.^[4]^ However, the exposure of fracture is less than that with the TTSFA.^[11]^ The deep and superficial fascia also need to be separated more widely, and the ulnar nerve still needs to be dissected. Therefore, there is still a large amount of soft tissue damage. This will prolong the operation time and increase the amount of bleeding. In addition, scar adhesion of soft tissue around joints easily occurs after surgery. It was found in clinical practice that there were still some patients who experienced the complication of elbow stiffness.^[23]^

In recent years, minimally invasive surgery has become widely adopted among surgeons, as it is associated with less tissue injury, early mobilization, rapid rehabilitation, and reduced postoperative mortality.^[24]^ Following this principle, the MLACMPA has been used, which is further improved on the basis of the BTBA. It has 2 advantages: The fracture site can be fully exposed. In particular, the carrying angle of the elbow joint is easy to change, thus preventing cubitus varus or cubitus valgus.^[25]^ When the MLACMPA is used during the operation, the bone crest at the lateral epicondyle of the humerus, the anterolateral side of the distal humerus, the olecranon fossa, the medial column and the lateral column can be exposed. The surgeon can reduce and fix the fracture under direct vision. With the short operation time, the advantage of this surgical approach in fracture exposure has been confirmed; Minor surgical trauma is the second advantage. In this study, the incision length in the MLACMPA group was 5.0 ± 0.6 cm, which was significantly <8.4 ± 0.9 cm in the TTSFA group and 9.3 ± 1.1 cm in the BTBA group. A small incision length can meet the aesthetic needs of patients. More importantly, in the MLACMPA group, we did not need to make an inverted V-shaped incision on the triceps tendon, free the ulnar nerve, or perform extensive separation between the deep and superficial fascia behind the elbow joint. At the elbow, the position of the ulnar nerve is very fixed in the ulnar nerve groove. The medial epicondyle of the humerus, the olecranon and the ulnar nerve groove can be easily distinguished by careful touching. There is no need to make an incision on the medial side of the elbow joint. Percutaneous Kirschner needling into the medial epicondyle of the humerus almost eliminates the risk of ulnar nerve injury.^[1]^ No ulnar nerve injuries were found in any case after the operation, which proved the advantages of this surgical approach. In this study, the ROM of the elbow in the MLACMPA group was significantly greater than that in the TTSFA group and the BTBA group at months 2, 4, and 6 postoperatively, indicating that elbow function recovered quickly after the operation in the MLACMPA group. In addition, the surgical bleeding in the MLACMPA group was significantly less than that in the TTSFA and BTBA groups. The results of a short incision, quick recovery of extension and flexion function of elbow and less surgical bleeding further proved that the operation involving the MLACMPA to fix supracondylar fracture of humerus with crossed Kirschner wire was more minimally invasive than the operations involving the TTSFA or the BTBA. In summary, the MLACMPA causes relatively less destruction to soft tissues but still provides enough exposure to reduce the fracture site.

Limitations of the MLACMPA included the following: First, when the child has an ulnar nerve injury, its exploration and repair cannot be realized by using this surgical approach; second, when the ulnar nerve is not exposed, the Kirschner wire is fixed at the internal condyle of the humerus, which may damage the ulnar nerve. This surgical approach cannot completely avoid the risk of ulnar nerve injury and is only suitable for surgeons with rich surgical experience. Third, due to the short follow-up time, it is unknown whether children developed elbow varus or valgus in the long term. Fourth, this cohort contained patients over a 20-year time span. Variables such as the level of surgeon experience, hospital setup, and rehabilitation program might affect the results. Nevertheless, we hypothesize that this study is meaningful. Additional comprehensive studies including longer evaluation periods and expanded sample sizes are needed.

This study is not a complete negation of the TTSFA and the BTBA. We hypothesize that the 2 approaches are still valuable. The biggest advantage of these surgical methods is that both sides of the fracture can be treated at the same time. In particular, the fracture can be more fully exposed with TTSFA, which is suitable for some severe comminuted fractures. In addition, the injured ulnar nerve can be explored and repaired.

## 5. Conclusion

The results of this retrospective analytical study indicate that compared to the TTSFA and the BTBA, using the MLACMPA for pin fixation in the treatment of irreducible displaced pediatric SHFs can result in better postoperative elbow function with less iatrogenic injury, which makes the approach worth popularizing in clinical practice.

## Author contributions

**Conceptualization:** Hu Da, Chang-Ming Xu.

**Data curation:** Hu Da, Liang Zhou, Qiao-Yun Xi.

**Formal analysis:** Hu Da, Liang Zhou, Chang-Ming Xu.

**Investigation:** Hu Da, Liang Zhou, Qiao-Yun Xi, Chang-Ming Xu.

**Methodology:** Hu Da, Liang Zhou.

**Project administration:** Hu Da, Chang-Ming Xu.

**Resources:** Qiao-Yun Xi, Chang-Ming Xu.

**Software:** Hu Da, Chang-Ming Xu.

**Supervision:** Chang-Ming Xu.

**Validation:** Chang-Ming Xu.

**Writing – original draft:** Hu Da, Liang Zhou, Chang-Ming Xu.
